# Potential Dual Role of Eugenol in Inhibiting Advanced Glycation End Products in Diabetes: Proteomic and Mechanistic Insights

**DOI:** 10.1038/srep18798

**Published:** 2016-01-07

**Authors:** Priyanka Singh, Ramesha H. Jayaramaiah, Sachin B. Agawane, Garikapati Vannuruswamy, Arvind M. Korwar, Atul Anand, Vitthal S. Dhaygude, Mahemud L. Shaikh, Rakesh S. Joshi, Ramanamurthy Boppana, Mahesh J. Kulkarni, Hirekodathakallu V. Thulasiram, Ashok P. Giri

**Affiliations:** 1 Division of Biochemical Sciences, CSIR-National Chemical Laboratory, Pune 411008, Maharashtra, India; 2 Division of Organic Chemistry, CSIR-National Chemical Laboratory, Pune 411008, Maharashtra, India; 3 Krantisinh Nana Patil College of Veterinary Science, Shirwal, Satara 412 801, Maharashtra, India; 4 National Centre for Cell Science, Pune 411007, Maharashtra, India; 5 Institute of Bioinformatics and Biotechnology, Savitribai Phule Pune University, Pune 411007, Maharashtra, India; 6 CSIR- Institute of Genomics and Integrative Biology, Mall Road, New Delhi 110007, India

## Abstract

Medicinally important genus *Ocimum* harbors a vast pool of chemically diverse metabolites. Current study aims at identifying anti-diabetic candidate compounds from *Ocimum* species. Major metabolites in *O*. *kilimandscharicum*, *O*. *tenuiflorum*, *O*. *gratissimum* were purified, characterized and evaluated for anti-glycation activity. *In vitro* inhibition of advanced glycation end products (AGEs) by eugenol was found to be highest. Preliminary biophysical analysis and blind docking studies to understand eugenol-albumin interaction indicated eugenol to possess strong binding affinity for surface exposed lysines. However, binding of eugenol to bovine serum albumin (BSA) did not result in significant change in secondary structure of protein. *In vivo* diabetic mice model studies with eugenol showed reduction in blood glucose levels by 38% likely due to inhibition of α-glucosidase while insulin and glycated hemoglobin levels remain unchanged. Western blotting using anti-AGE antibody and mass spectrometry detected notably fewer AGE modified peptides upon eugenol treatment both *in vivo* and *in vitro*. Histopathological examination revealed comparatively lesser lesions in eugenol-treated mice. Thus, we propose eugenol has dual mode of action in combating diabetes; it lowers blood glucose by inhibiting α-glucosidase and prevents AGE formation by binding to ε-amine group on lysine, protecting it from glycation, offering potential use in diabetic management.

Diabetes mellitus (DM) is a metabolic disorder of multiple etiologies characterized by elevated levels of blood glucose resulting from defects in insulin production, insulin action, or both. Pharmacological treatment of diabetes includes the use of oral anti-diabetic agents that aid in controlling hyperglycemia. These drugs either promote insulin secretion, insulin sensitivity; decrease the hepatic glucose output or aid in absorption of glucose. An important class of drug molecules, effective in management of diabetes is α-glucosidase inhibitors. These regulate blood glucose level by inhibiting digestion of oligosaccharides/carbohydrates like maltose, maltotriose, dextrins, sucrose etc. into glucose^1^. Examples of α-glucosidase inhibitors includes acarbose^2^, miglitol^3^, voglibose^4^ etc.

Apart from these drugs, inhibition of advanced glycation end products (AGEs) is considered as a useful therapeutic strategy in management of diabetes. AGEs are formed by a series of non-enzymatic reactions between reducing sugars and amine group of proteins^5^,^6^. Upon glycation, proteins tend to lose their structure and function^7^. AGEs bind to receptor for AGEs (RAGE), spawn reactive oxygen species, and downstream signaling contributes basically in elicitation of pro-inflammatory response^8^.

AGEs and AGE-RAGE axis has been implicated in various disease pathophysiologies including vascular and diabetic complications^5^,^6^.Growing evidences on involvement of AGEs in disease has made them attractive therapeutic targets. Thus molecule(s) that inhibit the formation of AGEs are effective in management of diabetes. Consequently extensive research effort has been devoted to develop anti-AGE therapeutics. These includes (i) preventing the formation of AGEs, example, ascorbic acid^9^, aspirin^10^, metformin^11^, etc. (ii) de-glycation of Schiff bases/Amadori products involving transglycation approach using drugs like hydralazine^12^, (iii) reversal of AGE induced modifications like cross links, example, phenacylthiazolium bromide (PTB)^13^, and (iv) preventing the body from ill effects of AGE formation, example, resveratrol and curcumin^14^,^15^. Combination of oral drugs, and oral drugs with insulin has been used for better control of diabetes and diabetic vascular complications. For example, combination therapy with repaglinide and rosiglitazone has been verified to be safe and effective in diabetes treatment^16^. Similarly, combination of voglibiose with glibenclamide or gliclazide^17^; or miglitol with metformin^18^ offer better glycemic control.

Therapeutically important genus *Ocimum*, comprising herbaceous members belonging to family Lamiaceae, is considered a boon for medicinal chemists^19^. Leaf extracts of several species have a long and successful history of being used in ancient folk medicine; having antioxidant^20^, antistress^21^, anticancer^22^, radiation protection^23^, antifungal^24^, insecticidal^25^ and several other bioactivities. The key to this medicinal potential of genus *Ocimum* might lie in the vast array of secondary metabolites and phytochemicals including terpenoids, phenylpropanoids, flavonoids, phenolic compounds etc. present in various plant parts. *Ocimum tenuiflorum* (former *O. sanctum*) leaf extracts have been shown to have hypoglycemic effects by induction of insulin secretion from perfused pancreas, isolated islets and clonal pancreatic β-cells^26^,^27^; however, the principle compound and its mechanism of action are poorly understood. In view of this, we demonstrate the anti-diabetic activity of eugenol from *O. gratissimum* using *in vitro* and *in vivo* approaches. Here we have shown, eugenol isolated from *O. gratissimum* to have a potential dual effector role in diabetes control; it acts as an effective α-glucosidase inhibitor as well as a glycation inhibitor, mimicking the effect of combination therapy. Although both synthetic and natural anti-diabetic therapeutics are available, latter seems to be the obvious choice owing to its low toxicity and lesser side effects. Thus, identifying anti-AGE lead molecules of natural origin would provide a significant thrust to diabetes research in future.

## Results and Discussion

### Chemical profiling unravels terpene and phenylpropanoid abundance in *Ocimum* species

GC-MS based chemical profiling of leaf tissue of three *Ocimum spp.* revealed that each species was rich in a specific set of compounds representing a distinct metabolic fingerprint. Results indicated predominance of monoterpenes, sesquiterpenes and phenylpropanoids. Hydrocarbons including dodecane, dodecene, heptene, octane derivatives etc. were detected in minor quantities. The entire list of compounds identified is provided in Supplementary Table S1 online. Table 1 provides a selected subset of major metabolites screened for antiglycation activity using BSA-AGE assay.

### *In vitro* inhibition of AGEs by metabolites from *Ocimum* species

**Figure 1:**
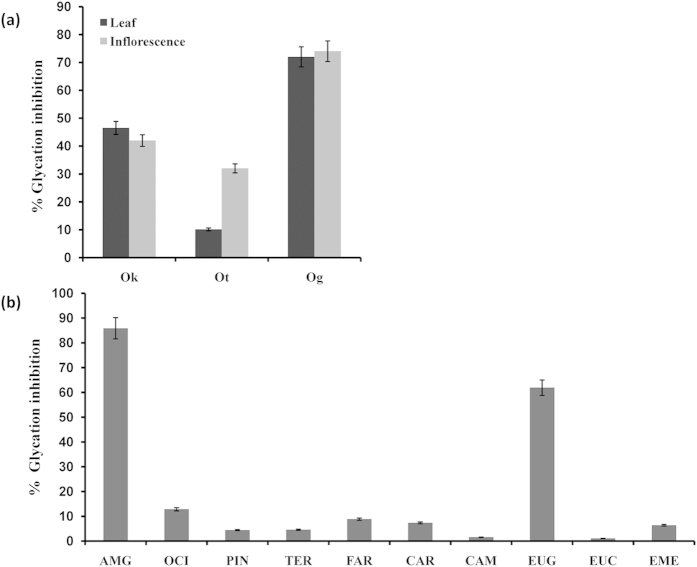
*In vitro* BSA-AGE inhibition assay. Glycation inhibition shown by (**a**) leaf and inflorescence extracts of *O. kilimandscharicum* (Ok), *O. tenuiflorum* (Ot), *O. gratissimum* (Og) and (**b**) standard compounds, aminoguanidine (AMG), ocimene (OCI), pinene (PIN), terpinolene (TER), farnesene (FAR), β- caryophyllene (CAR), camphor (CAM), eugenol (EUG), eucalyptol (EUC) and eugenol methyl ether (EME).

**Figure 2:**
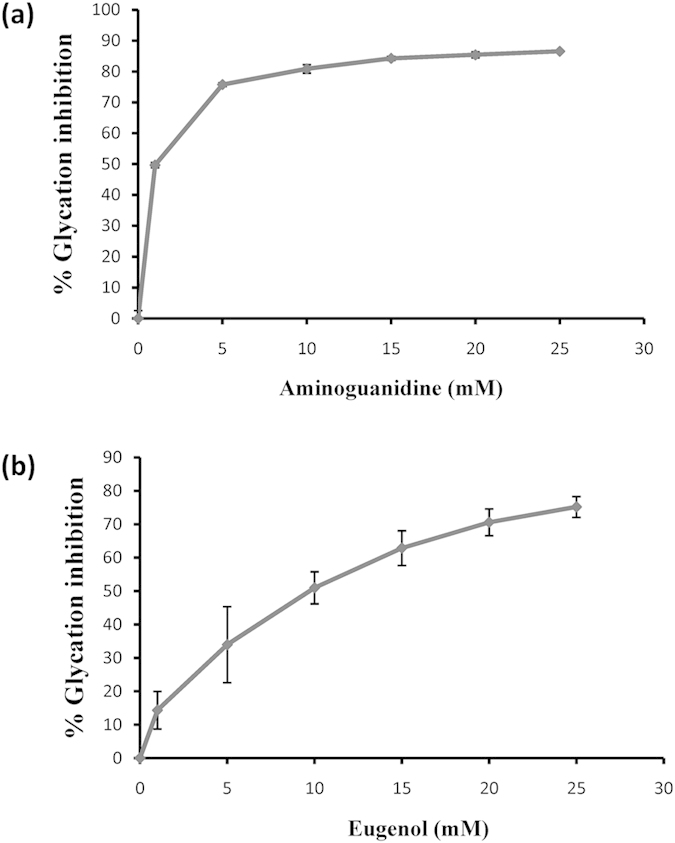
Glycation inhibition assays by aminoguanidine and eugenol. IC_50_ values for AGE inhibition by (**a**) Aminoguanidine and (**b**) Eugenol. Values represent mean ± standard deviation (n = 3).

Leaf and inflorescence extracts from three *Ocimum* species and major metabolites therein including camphor, eucalyptol, eugenol, eugenol methyl ether (EME), ocimene, α-pinene, terpinolene, β-caryophyllene and farnesene were evaluated for their *in vitro* anti-glycation activity using BSA-AGE fluorescence assay. Maximum inhibition of glycation was observed with *O. gratissimum* extract, which is rich in eugenol. Inflorescence and leaf extracts of *O. gratissimum* inhibited the formation of AGEs by 74% and 72%, respectively (Fig. 1a). *O. tenuiflorum* leaf extracts rich in EME showed least (10%) inhibition of glycation (Fig. 1a). *O. kilimandscharicum* leaf and inflorescence extracts, rich in camphor and eucalyptol (Supplementary Table S1) displayed significant inhibition of AGE formation, 46% and 42%, respectively (Fig. 1a). Of all the metabolites assessed, eugenol displayed highest, 58% inhibition of glycation (Fig. 1b). Other metabolites did not inhibit AGE formation significantly. Based on these studies, eugenol, the major metabolite present in *O. gratissimum* was considered for *in vivo* studies. Inhibitory concentration required to inhibit 50% AGE formation (IC_50_) for eugenol was 10 mM (Fig. 2b) while for aminoguanidine hydrochloride was 1 mM (Fig. 2a).

Since anti-glycation activity of extracts was significantly higher than that of individual metabolites, it was hypothesized that either of the following possibilities might exist: (i) anti-glycation activity of tissue extracts maybe due to synergistic action of a group of metabolites rather than a single metabolite or (ii) the metabolites present *in planta* are structurally modified, imparting them enhanced anti-glycation potential. To test this hypothesis, major metabolites (eugenol, EME and camphor) were purified and characterized by NMR analysis. Results indicated that there were no structural difference between metabolites present *in planta*, in comparison with standard compounds procured from Sigma-Aldrich (Supplementary Table S2). Since these metabolites present *in planta* were not structurally modified, it can be suggested that the increased antiglycation activity of *Ocimum* tissue extracts is probably due to synergistic effect of a group of metabolites. However, further investigation is required to acertain the hypothesis.

### Eugenol shows increased binding affinity for surface lysine residues on mouse serum albumin but does not alter the protein secondary structure

**Figure 3:**
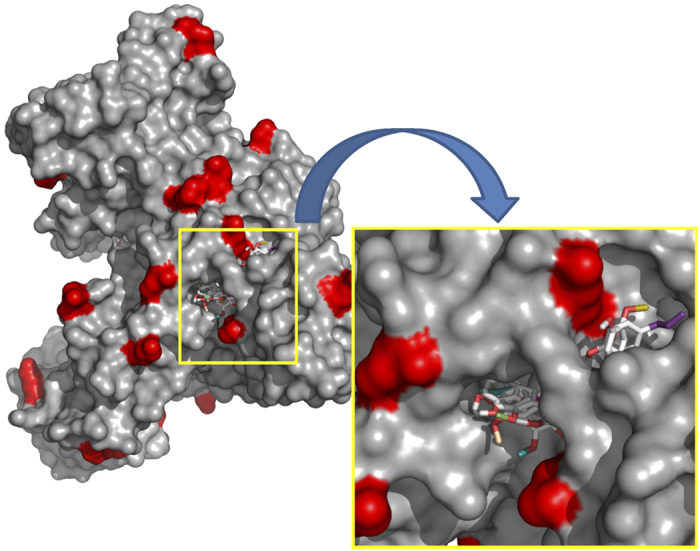
*In silico* analysis of interactions between eugenol and MSA. Blind docking and probablity analysis of eugenol with MSA. Surface exposed lysine have been marked in red. Inset depicts binding of several eugenol molecules to surface lysine residues on MSA.

Based on *in vitro* BSA-AGE assay, eugenol was observed to be a potent inhibitor of AGEs compared to EME. Structurally, the difference between eugenol and EME is that, in eugenol a hydroxyl group is present at the para position (4′-OH); however, in EME this group is masked by the presence of a methyl group. We speculate the anti-glycation activity of eugenol might be due to the presence of free 4′-OH group. Previous studies support the fact that, presence and position of hydroxyl group determines the activity of flavonoids^28^. We imply that masking of active hydroxyl group in EME is responsible for decline in antiglycation activity of the compound. To gain more insight into eugenol-protein interaction, blind docking study of eugenol with mouse serum albumin (MSA) was performed. Out of many surface-exposed lysine, blind docking results indicate that eugenol preferentially binds to Lys-236 and Lys-375. In Fig. 3, surface exposed lysine residues have been marked in red, and binding of multiple eugenol molecules to single MSA molecule is depicted. Eugenol shows stronger binding (average binding energy, 6 Kcal/mol) with surface exposed lysines as compared to aminoguanidine hydrochloride (average binding energy, 4.3 Kcal/mol). EME also showed strong binding affinity for MSA (Supplementary Fig. S1), however, it did not display good inhibition of glycation *in vitro*. Thus, preliminary evidence suggests that the 4′ -OH group of eugenol is potentially capable of binding to the amine group of lysine residues on protein molecule and competitively inhibiting the binding of sugar.

**Figure 4:**
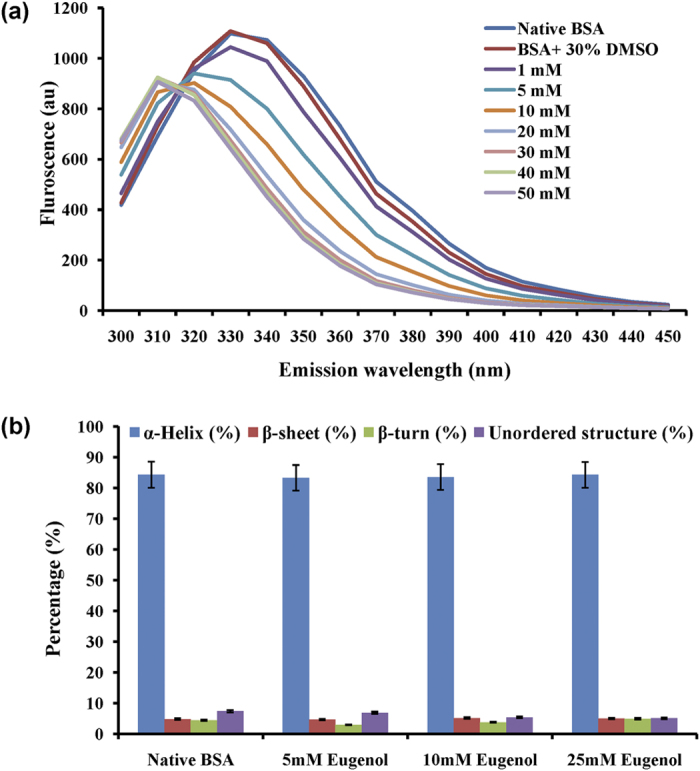
Biophysical analysis of BSA and eugenol interaction (**a**) Fluorescence quenching of BSA by eugenol (**b**) CDPro analysis of native BSA and BSA treated with 5, 10 and 25 mM eugenol.

Intrinsic fluorescence assay and circular dichorism (CD) were performed to understand the nature of interaction between eugenol and BSA. In the intrinsic fluorescence assay, it was evident that eugenol binds to BSA in a concentration dependent manner (Fig. 4a). As the concentration of eugenol was increased, a consistent decrease in intrinsic fluorescence intensity was documented. CD of BSA incubated with eugenol was performed to understand the effect of binding of eugenol on the secondary structure of BSA. However, the secondary structure of BSA (Fig. 4b) remained unchanged (84.3 ± 1.9%, α-helix; 4.85 ± 0.2%, β-sheet; 4.45 ± 1.9%, β-turn; 7.4 ± 0.2% unordered). The interaction did not induce any transition from α-helix to β-sheet or *vice versa*. in the structure of protein. Hence we conclude that eugenol binds to BSA but the binding does not cause any significant change in the secondary structure of BSA.

### Eugenol administration affects blood biochemical parameters

**Figure 5:**
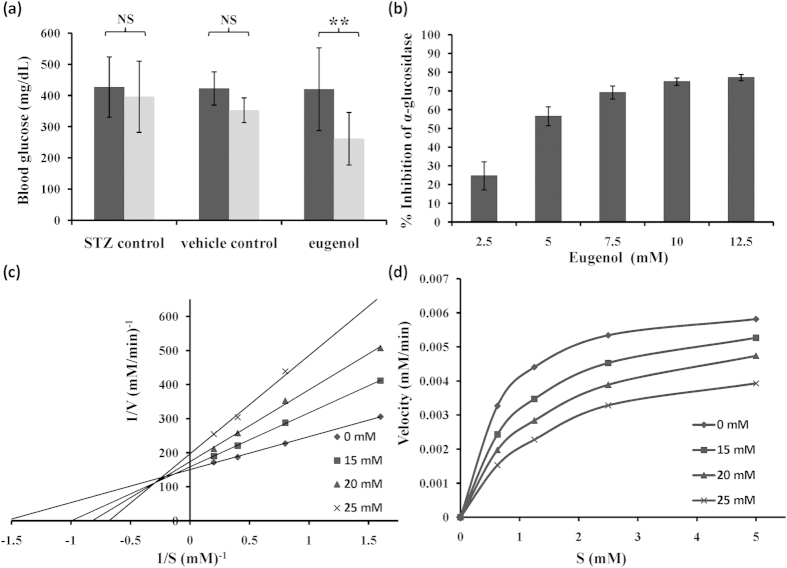
Kinetic studies of alpha-glucosidase inhibition by eugenol (**a**) Blood glucose measurement after intraperitoneal administration of eugenol (n = 8) in STZ- induced balb/c mice. Unpaired t-test suggested significant differences between data at p < 0.01(indicated as ‘**’) and p < 0.05 (indicated as ‘*’). NS represents non-significant difference in data. (**b**) α-glucosidase inhibition assay. Inhibition kinetics depicted via (**c**) Lineweaver-burke plot and (**d**) Michaelis- Menten plot showing mixed inhibition of α-glucosidase by eugenol.

**Figure 6:**
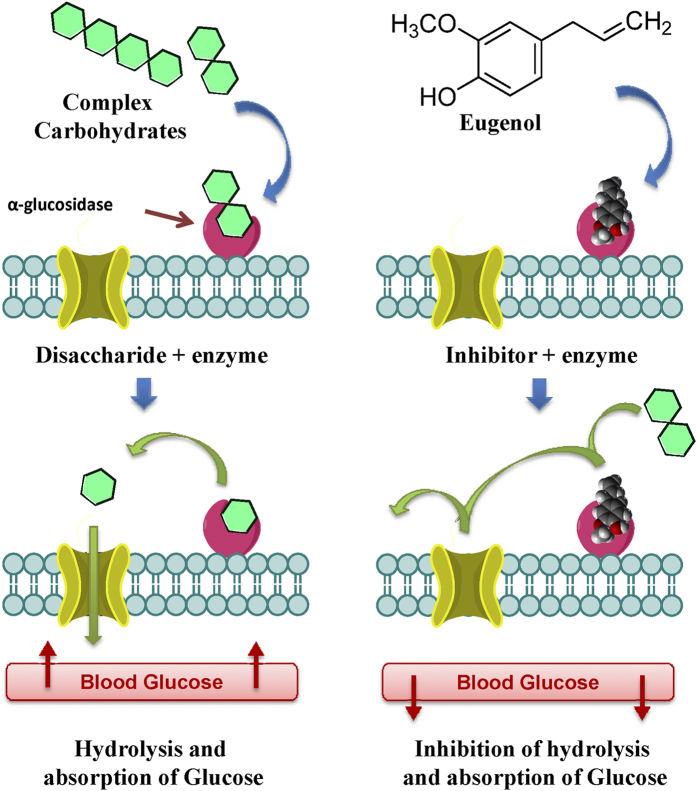
Inhibition of α-glucosidase by eugenol slows carbohydrate metabolism resulting in decrease in blood glucose.

The effect of administration of eugenol on blood glucose, HbA1c and insulin was investigated. Since hyperglycemia is known to be the foremost cause of diabetic complications, molecule(s) that lower blood glucose are frontrunners for management of diabetes. Mice belonging to Group I (STZ control) and group II (vehicle control) did not show any significant decrease in blood glucose (396 mg/dL and 353 mg/dL, respectively). Mice belonging to Group III (eugenol- treated mice) exhibited a 38% decrease in blood glucose, with levels dropping from 420 mg/dL to 262 mg/dL, on an average (two-tailed *p-value*, 0.0042). Lowering blood glucose level helps subsequently in lowering formation of AGEs. Administration of eugenol showed significant decrease in blood glucose level (Fig. 5a). Although the blood glucose levels decreased, it did not lead to a severe hypoglycemic condition. Decrease in blood glucose may be attributed to the inhibition of α-glucosidase activity (Fig. 6), as discussed in subsequent section. Furthermore, the blood glucose levels increased when the treatment was withdrawn.

**Figure 7:**
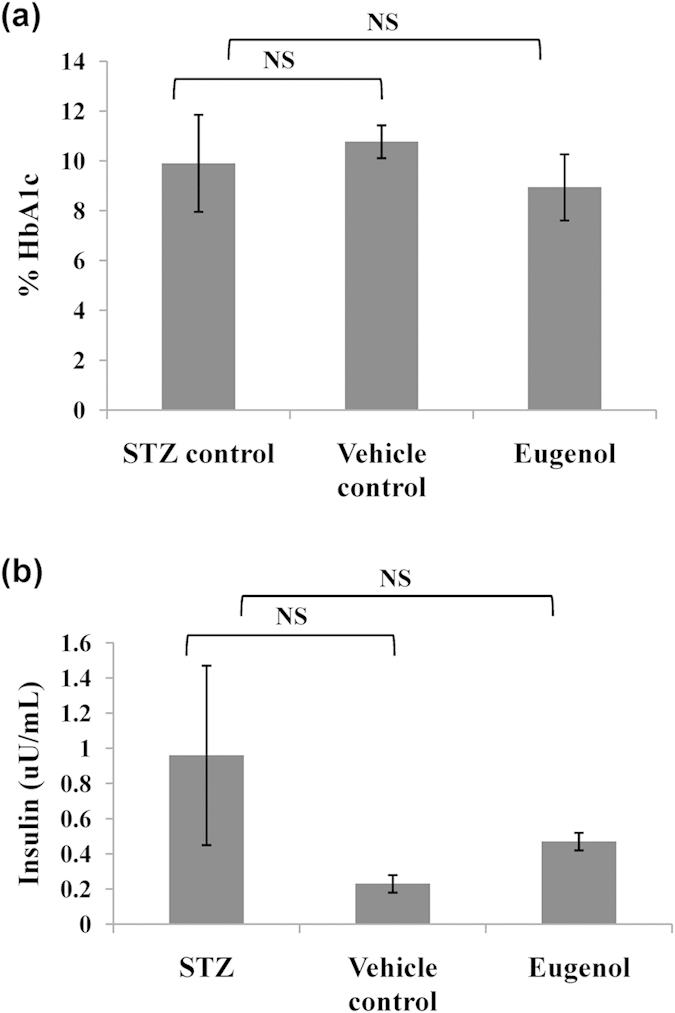
Analysis of blood biochemical parameters. Measurement of (**a**) HbA1c, (**b**) plasma insulin. Unpaired t-test suggested significant differences between data at *p* < 0.05 (indicated as ‘*’). NS represents non-significant difference in data.

HbA1c essentially serves as a marker for monitoring glycemic status over a period of three months. We did not notice any significant change in HbA1c levels between different groups of mice: Group I (9.9), Group II (10.7), Group III (8.9), values in the parenthesis represent average for each group of treatment (Fig. 7a). The possible reason behind no significant change in HbA1c level may be due to the short duration of study (45 days). HbA1c levels are known to changes over a period of 3 to 4 months. Since our study was restricted to a short period of 45 days, no drastic changes in HbA1c levels were observed. No significant change in the insulin level was evident between the groups (Fig. 7b) suggesting that eugenol lowers the blood glucose by inhibiting glucosidase activity.

### Mixed inhibition of α-glucosidase by eugenol might lead to decrease in blood glucose

We speculate that, the decrease in blood glucose may be due to inhibition of intestinal α-glucosidase. α-Glucosidase is localized in brush border epithelium of small intestine and catalyzes the conversion of oligosaccharides/carbohydrates like maltose, maltotriose, dextrins, sucrose etc. into glucose. α-Glucosidase inhibitors are known to lower blood glucose level by slowing carbohydrate metabolism^2^,^3^,^4^. Eugenol inhibited yeast α-glucosidase in a concentration dependent manner (Fig. 5b) with IC_50_ value around 5 mM. Lineweaver-Burke plot analysis revealed mixed type of inhibition; thus, eugenol can bind to both enzyme and enzyme-substrate complex. Figure 6 shows a schematic representation of inhibition of α-glucosidase by eugenol. Therefore, eugenol as an α-glucosidase inhibitor can be a potential candidate in the treatment and management of diabetes.

### Eugenol treated mice display significantly less histopathological lesions

**Figure 8:**
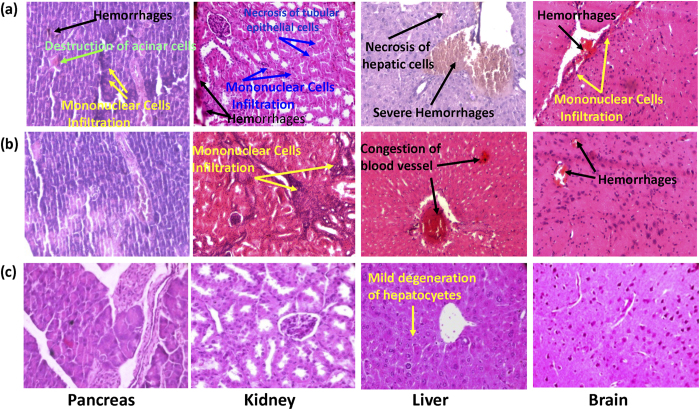
Histopathological examination of pancreas, kidney, liver and brain tissues of (**a**) STZ control (**b**) vehicle control (**c**) eugenol treated mice. Arrows indicate histopathological lesions in respective tissues.

Histopathology was performed to understand the gross and microscopic effect of eugenol on different tissues (spleen, liver, heart, lungs, kidney, pancreas and brain). Spleen, heart and lung tissues of mice belonging to all three groups did not show significant abnormal lesions. However, brain, pancreas, kidney and liver tissue displayed moderate to severe histopathological lesions depending upon the treatment administered. Mice belonging to Group I revealed most severe lesions (Fig. 8a) followed by those belonging to Group II (Fig. 8b). In both these groups, brain tissue revealed mononuclear cell proliferation and focal hemorrhages. Pancreas exhibited focal destruction of acinar cells, hemorrhages and mononuclear cell infiltration. Kidney sectioning exposed necrosis of tubular epithelial cells, focal hemorrhages and mononuclear cell infiltration. Liver reflected severe hemorrhages and focal necrosis of hepatic cells. Occurrence of these severe lesions may be due to oxidative stress related cell injury caused by hyperglycemia in these groups. Occurrence of aforementioned lesions was significantly less in mice belonging to Group III (Fig. 8c), however, mild degeneration was observed in hepatocytes. The results indicate that eugenol treated mice displayed healthier histopathology than diabetic mice emphasizing therapeutic effect of eugenol against hyperglycemia.

### Western blot analysis shows *in vitro* and *in vivo* inhibition of AGEs by eugenol

**Figure 9:**
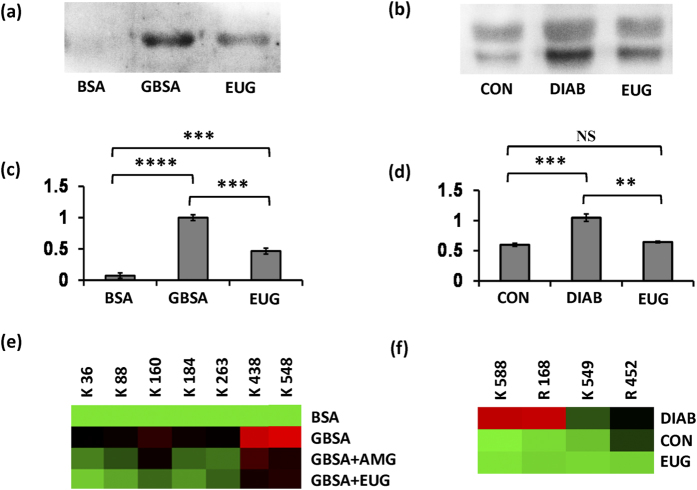
Proteomic analysis of *in vitro* and *in vivo* samples for AGE formation. Western blot using anti-AGE antibody & blot density analysis of *in vitro* BSA-AGE assay samples (**a**,**c**) and *in vivo* plasma samples (**b**,**d**) for probing AGE formation. One way ANOVA followed by unpaired t-test suggested significant differences between data at *p* < 0.01 (indicated as ‘**’), *p* < 0.001 (indicated as ‘***’) and *p* < 0.0001 (indicated as ‘****’). NS represents non-significant difference in data. Heat map showing extent of AGE induced modifications on specific lysine and arginine residues in (**e**) *in vitro* BSA-AGE assay and (**f**) plasma protein, identified by LC-MS^E^. Heat map generated using Multi Experiment Viewer (MEV) software. (GBSA, glycated BSA; AMG, aminoguanidine; EUG, eugenol-treated sample; CON, control healthy mice; DIAB, STZ control plasma).

Western blot analysis of *in vitro* BSA-AGE assay samples and *in vivo* plasma samples showed significant differences in eugenol-treated and un-treated glycated BSA (Fig. 9a,c) as well as eugenol-treated and un-treated STZ mice plasma (Fig. 9b,d) following statistical analysis of blot density data. The results clearly indicate significant reduction in binding of glucose in presence of eugenol, showing western blot relative density, 0.46 for eugenol-treated glycated BSA compared to control with *p* = 0.0008 (*p* < 0.001 indicated by ***) in BSA-AGE samples (Fig. 9c) and 0.64 for eugenol-treated STZ mice with *p* = 0.0011 (*p* < 0.01 indicated by **) in *in vivo* plasma samples (Fig. 9d).

One of the reasons for lesser accumulation of AGEs in plasma could be because of decreased level of blood glucose, which can be attributed to α-glucosidase inhibitory activity of eugenol. Further, eugenol can also inhibit the formation of AGEs by competitively inhibiting the binding of sugar to proteins both *in vivo* and *in vitro,* as discussed previously. Thus, both these factors result in overall lesser accumulation of AGEs.

### LC-MS analysis reveals lesser extent of AGE modification on peptides upon eugenol treatment

LC-MS analysis was performed to understand the sites of AGE modification for both *in vitro* BSA-AGE assay and *in vivo* plasma protein especially mouse serum albumin (MSA). Heatmap analysis showing extent of AGE modification on glycated peptides, including glycation sensitive amino acid residues (GSAR) containing peptides of MSA is depicted in Fig. 9f. It was noticed that AGE modified peptides were found to decrease in response to eugenol treatment and were found to be highest in plasma of STZ treated diabetic mice. The MS/MS spectra of AGE modified peptides are provided in Supplementary Table S3 and Supplemetary Fig. S2. Glycation sensitive sites R168 and R452^12^,^29^, which are suggested to be markers for type II diabetes^29^ significantly showed lesser extent of glycation in response to eugenol treatment. The fact that extent of AGE-modification is less on peptides containing GSAR residues, makes eugenol a convincing candidate for early inhibition of glycation. Similarly, eugenol showed significantly lesser AGE modification in *in vitro* glycated BSA sites K36, K88, K160, K184, K263, K438 and K548 in comparison with glycated BSA and positive control AMG (Fig. 9e). The MS/MS spectra for these sites are provided in Supplementary Table S4 and Supplementary Fig. S3). Thus, LC-MS analysis of glycated peptides reveals that eugenol, contributes significantly in reducing the extent of glycation, both *in vitro* and *in vivo* eventually resulting in lesser accumulation of AGEs.

## Conclusions

**Figure 10:**
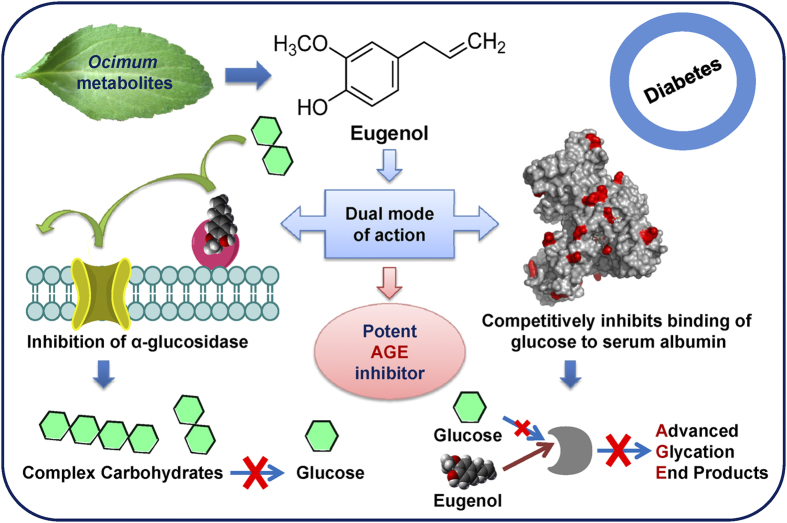
Schematic presentation of proposed potential dual role of eugenol in inhibiting AGEs. Leaf photograph courtesy, R.H.J. (co-author).

We identified eugenol, a phenylpropanoid, as a potent inhibitor of AGEs both by *in vitro* and *in vivo* studies. We propose that eugenol exerts potential dual mode of action in combating AGEs (Fig. 10). It might inhibit intestinal α-glucosidase and block the conversion of complex carbohydrates to glucose, resulting in lower blood glucose level and subsequent reduction in AGE formation. Also, eugenol competitively inhibits the binding of sugar to serum albumin by binding to amine group of surface exposed lysine residues *via* its reactive 4′-OH group. The above observations were strongly supported by biophysical, biochemical, proteomic and histopathological studies. Thus, here we report eugenol, isolated from *O. gratissimum*, as a natural, FDA-approved non-toxic potent inhibitor of AGEs that can be used in management of diabetes.

## Materials and Method

### Chemicals and plant material

All chemicals were procured from Sigma-Aldrich (St Louis, MO, USA), otherwise mentioned. Yeast α-glucosidase and *p*-nitrophenyl-α-D-glucopyranoside were procured from SRL and Himedia (Mumbai, MS, India), respectively. Three species namely *O. kilimandscharicum*, *O. tenuiflorum* and *O. gratissimum* were grown under the following greenhouse conditions: temperature, 28 to 30 °C; humidity, 35 to 40%; light conditions, 16 h light, 8 h dark. After harvesting, tissue was immediately subjected to further extraction procedure.

### Gas chromatography-mass spectrometry (GC-MS) analysis of *Ocimum* plant tissues

Extractions were performed as described earlier^25^. Leaf tissue (1 g) was mixed in 10 mL dichloromethane (DCM) and kept for 18 to 24 h at 28 °C. The extract was filtered and incubated for 2 h at −20 °C to allow lipid precipitation. DCM extract was filtered again, concentrated under vacuum on a rotary evaporator and subjected to GC and GC-MS analyses as reported previously^25^.

### Purification and NMR characterization of major metabolites from *Ocimum* species

Large-scale metabolite extraction was performed using 10 g leaves as mentioned earlier^25^. Thin-layer chromatography (TLC) was performed on silica gel G-coated plates (0.25 mm for analytical) developed three times in 5% petroleum ether in ethyl acetate. Compounds were visualized under UV light (254 nm) or by spraying with a solution of 3% anisaldehyde, 2.8% H_2_SO_4_, 2% acetic acid in ethanol followed by heating for 1 to 2 min. Purification of major compounds was performed by flash chromatography using 240–400 mesh silica gel columns and petroleum ether-ethyl acetate gradient mixture as the eluent.

NMR (^1^H and ^13^C) for purified compounds was carried out on Bruker DRX-500 (500 MHz), Bruker AC-200 (200 MHz) spectrometers in CDCl_3_. Chemical shifts were reported in parts per million, with respect to tetramethylsilane as the internal standard.

### BSA-AGE fluorescence assay

Stock solutions (100 mM) of aminoguanidine hydrochloride, ocimene, α-pinene, terpinolene, farnesene, β-caryophyllene, camphor, eugenol, eugenol methyl ether (EME) and eucalyptol were prepared in 30% DMSO and vortexed for 15 min for uniform mixing. For extract preparation, dichloromethane extract was concentrated to dryness under vacuum on a rotary evaporator, re-dissolved in 30% DMSO and vortexed for 15 min.

The reaction was set up as described earlier^30^. BSA glycation reaction was carried out by incubating 1 mL of 50 mg mL^−1^ BSA in 0.1M phosphate buffer (pH 7.4) and 0.5M dextrose monohydrate containing 5 mM sodium azide as bacteriostat at 37 °C for 7 days with extracts and 15 mM of above mentioned compounds. DMSO (30%) and aminoguanidine were used as solvent and positive control, respectively. The BSA glycation was monitored at 370/440 nm by using Varioskan Flash 4.00.53 spectrofluorometer (Thermo Scientific, Waltham, MA, USA). Percent inhibition of glycation was calculated by using the formulae; (C-T)/C × 100 where C is the relative fluorescence intensity of glycated BSA in absence of an inhibitor and T is the relative fluorescence intensity of glycated BSA in presence of an inhibitor.

### Blind docking and probability analysis

Blind docking and probability analysis of eugenol with mouse serum albumin (MSA) was performed as described earlier^31^.

### Intrinsic fluorescence assay

BSA (50 mg mL^−1^) in phosphate buffer (50 mM, pH 7.4) was incubated at 37 °C for 2 h with different concentrations of eugenol (1–50 mM) dissolved in 30% DMSO. Intrinsic fluorescence was monitored using spectrofluorometer (excitation: 280 nm, emission: 300–450 nm).

### Circular dichorism analysis of BSA and BSA-eugenol complexes

BSA (50 mg mL^−1^) in phosphate buffer (50 mM, pH 7.4) was incubated at 37 °C for 2 h with varying concentrations of eugenol (5, 10 and 25 mM) dissolved in 30% DMSO. 0.02 mg mL^−1^ concentration of protein was used to measure the CD spectra. All the CD spectra were recorded at room temperature (24 °C) using JASCO J-815 CD spectropolarimeter (Jasco Inc., Easton, MD, USA) over wavelength ranging from 250-190 nm.

### Animal experiments

*Ethics statement*: All animal experiments were approved by Institutional Animal Ethics Committee of National Centre for Cell Sciences, Pune, MS, India. The experimental protocols were carried out in accordance with the guidelines of Committee for the Purpose of Control and Supervision of Experiments on Animals, India

Twenty healthy male balb/c mice, 6 to 8 weeks old, weighing 20 to 25 g were used for experiments. Mice were maintained in standard polyvinyl cages under the following conditions: temperature, 24 to 26 °C; humidity, 35 to 40%; light conditions, 16 h light, 8 h dark and fed on pellet diet and water *ad libitum*. Streptozotocin (STZ; 45 mg/kg body weight, 100 μL) was administered intraperitoneally in citrate buffer (50 mM, pH 4.5) to mice for 5 consecutive days to induce diabetes. Mice were monitored for a period of 15 days for establishment of stable hyperglycemic condition. During this time parameters like weight, water intake, physical appearance, animal behavior, urine output and blood glucose were measured routinely. Mice which displayed stable hyperglycemic condition were chosen for experiments and divided in three groups: Group I, STZ control; Group II, Vehicle control; Group III, eugenol- treated mice. 3 mice per control group and 9 mice per treated group were taken. 100 μL intraperitoneal injection of eugenol (100 mg/kg body weight) in vehicle (Ethanol: Tween80: Saline = 1:1:18) twice a week for two weeks was administered. Time between consecutive injections was 3 days.

### Estimation of blood glucose and HbA1c levels

Blood glucose and HbA1c levels were measured using Bayer’s CONTOUR blood glucose meter and Bayer A1C Now Kit (Bayer, Leverkusen, NRW, Germany), respectively according to the manufacturer’s instructions.

### α-glucosidase inhibition assay and kinetics

α-glucosidase inhibition assay was performed as described earlier^32^. 100 μL eugenol in varying concentration (2.5 to 12.5 mM; prepared in 30% DMSO), 50 μL of 5 mM *p*-nitrophenyl- α-D-glucopyranoside (PNPG) and 50 μL yeast α-glucosidase (0.25 U/mL) were mixed and incubated at 37 °C for 30 min. Reaction was terminated by addition of 2 mL of 200 mM Na_2_CO_3_. Amount of *p*-nitrophenol released was measured using a spectrophotometer at 405 nm. Mode of inhibition of yeast α-glucosidase by eugenol was determined by measuring enzyme activity with increasing concentration of PNPG in the presence and absence of eugenol at different concentration^32^. Type of enzyme inhibition was determined using Lineweaver-burke plot analysis using Michelis-Menten kinetics.

### Plasma collection and insulin measurement

Plasma was collected on the last day of experiment (day 45) and stored at −80 °C until further use. Plasma insulin measurements were performed at Department of Biochemistry, King Edward Memorial (KEM) Hospital, Pune, MS, India.

### Tissue processing for histopathology

Mice were sacrificed on the last day of experiment (day 45) by cervical dislocation. Part of spleen, liver, heart, lungs, kidney, pancreas and brain were fixed in 10% formalin for histopathological analysis. Tissues were processed in a Leica TP 1020 tissue processor and embedded in paraffin blocks using Leica EG 1160 paraffin embedder. The paraffin blocks were cut into sections of 4 mm using a Microm HM 360 microtome. The slides were stained with hemotoxylin and eosin using a Microm HMS-70 stainer. Permanent slides were made and evaluated for histopathological changes under Olympus BX51 microscope.

### Western blotting

Western blotting was performed in biological duplicates and technical triplicates for plasma samples. *In vitro* BSA-AGE assay was performed in duplicate and technical triplicates of each sample. Protein (5 μg), in each case, was resolved on 12% SDS-PAGE, transferred onto polyvinylidene difluoride membrane (PVDF) membranes and blocked overnight at 4 °C with 5% membrane blocking agent prepared in TBS. The membranes were incubated with primary antibody Anti-AGE (Abcam) at a dilution of 1:2000 for 1 h, followed by 1:5000 secondary antibody (Goat Anti-Rabbit IgG) conjugated with horseradish peroxidase (HRP) for 30 min. Protein bands were visualized using WesternBright ECL HRP substrate (Advansta, Menlo Park, CA, USA) and documented by using Syngene DYVERSITY gel doc system (Syngene, Cambridge, UK).

### In-gel trypsin digestion and LC-MS analysis of *in vitro* samples

In gel trypsin digestion was performed a described earlier^33^. Tryptic peptides were analyzed by nano LC-MS^E^ (MS at elevated energy) using a Nano Acquity UPLC system (Waters Corporation, Milford, MA) online coupled to a Q-TOF, SYNAPT-HDMS (Waters Corporation) as described by Cheng *et al.*^34^ LC-MS^E^ data were processed using Protein Lynx Global Server 2.4 (PLGS; Waters Corporation). Search was performed against UniProt-P02769 (BSA) sequence database. Glycation modifications of lysine, amadori (+ 162.05Da), CML (+58.0Da) and CEL (+72.02Da) were considered as additional variable modifications. Glycation modifications identified by PLGS were manually validated as described by Bhonsle *et al.*^35^,^36^

### In-solution trypsin digestion and LC-MS/MS analysis of plasma proteins

In solution trypsin digestion was performed as described earlier^33^. Peptides were desalted using Zip tip C18 (Millipore, Billerica, MA, USA), concentrated by vacuum centrifuge and stored at −20 °C until further use. Peptides (5 μl injections containing 3.5 μg of peptides) were loaded on Eskigent C18 reverse phase column (100*0.3 mm, 3 μm, 120 Å) with 97% of mobile phase A (100% water, 0.1% formic acid) and 3% of mobile phase B (100% acetonitrile, 0.1% formic acid) at 8 μl/min flow rate. The peptides were separated at 8 μl/min flow rate for 100 min linear gradient of 3% to 50% mobile phase B. After 100 min the gradient was raised to 90% B for 9 min and the column was re-equilibrated to 3% mobile phase B for 11 min. All samples were analyzed on Triple TOF 5600 mass spectrometer (Sciex; Concord, ON, Canada) as described by Jones *et al.*^37^ The samples were acquired in positive and high-sensitivity mode using Electrospray ionization (ESI) method. The acquired MS dataset was processed using the Proteome Discoverer software (Version 1.4.1.14, Thermo Fisher Scientific, Bremen, Germany). SEQUEST HT search engine was used for peptide identification. Data was searched against UniProt P07724 (mouse serum albumin) sequence database. Ion search parameters used included peptide precursor and fragment mass tolerance- 10 ppm and 0.5 Da respectively with 2 missed cleavages and 1% FDR. Glycation modifications of lysine, Amadori (162.05 Da), CML (+58.0 Da) and CEL (+72.02 Da) were considered as additional variable modifications.

### Statistical Analysis. 

GC-MS analyses of leaf tissue, *in vitro* BSA-AGE assay/s for extracts and compounds and IC_50_ assays for eugenol and aminoguanidine were performed in triplicates and values were represented as mean ± standard deviation. Unpaired t-test was performed for blood glucose, plasma insulin and HbA1c measurements. Western blotting for plasma samples was performed in biological duplicates and technical triplicates. One-way ANOVA followed by unpaired t-test was performed for blot density analysis. Unpaired t-test suggested significant differences between data at *p* < 0.0001 (indicated as ‘****’), *p* < 0.001 (indicated as ‘***’), *p* < 0.01 (indicated as ‘**’) and *p* < 0.05 (indicated as ‘*’). NS represents non-significant difference in data.

## Additional Information

**How to cite this article**: Singh, P. *et al.* Potential Dual Role of Eugenol in Inhibiting Advanced Glycation End Products in Diabetes: Proteomic and Mechanistic Insights. *Sci. Rep.* **6**, 18798; doi: 10.1038/srep18798 (2016).

## Supplementary Material

Supplementary Information

## Figures and Tables

**Table 1:**
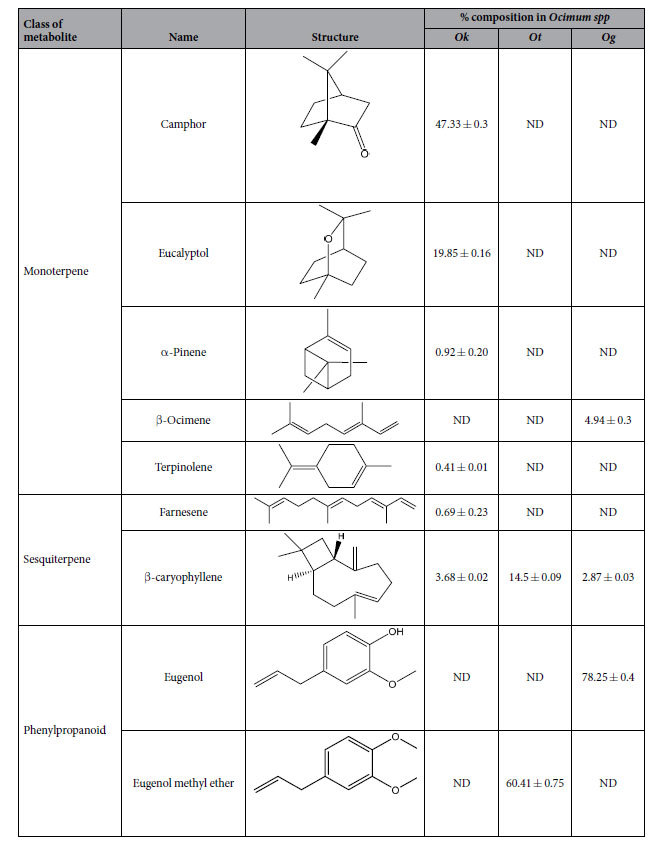
*Ocimum spp.* metabolites screened for antiglycation activity using BSA-AGE assay.

^ND (not detected), *Ok* (*O. kilimandscharicum*), *Ot* (*O. tenuiflorum*), *Og* (*O. gratissimum*).
