# Supplementary material for: Potential Dual Role of Eugenol in Inhibiting Advanced Glycation End Products in Diabetes: Proteomic and Mechanistic Insights
Source: Sci Rep. 2016 Jan 7;6:18798. doi: 10.1038/srep18798 (PMC4704049; doi:10.1038/srep18798)
Supplement: Supplementary Information [file srep18798-s1.pdf]

SUPPORTING INFORMATION

Potential Dual Role of Eugenol in Inhibiting Advanced Glycation End Products in Diabetes: Proteomic and Mechanistic Insights

Priyanka Singh[1, 2], Ramesha H. Jayaramaiah[1], Sachin B. Agawane[1], Garikapati Vannuruswamy[1], Arvind M. Korwar[1], Atul Anand[1,2], Vitthal S. Dhaygude[3], Mahemud L. Shaikh[4], Rakesh S. Joshi[5], Ramanamurthy Boppana[4], Mahesh J. Kulkarni[1], Hirekodathakallu V. Thulasiram[2, 6], Ashok P. Giri[1,*]

Table of contents: **Page No.**

Supplementary Figure S1……………………………………………….S2

Supplementary Figure S2……………………………………………….S3

Supplementary Figure S3……………………………………………….S4-S5

Supplementary Table S1……………………………………………….. S6

Supplementary Table S2……………………..………………………S7

Supplementary Table S3…………………………………………….......S8

Supplementary Table S4…………………………………………….......S8

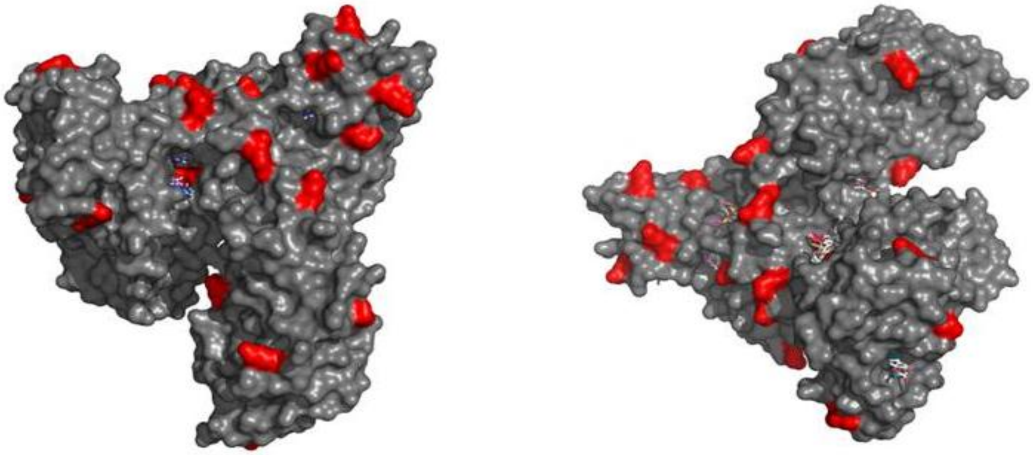

(A) (B)

Supplementary Figure S1: Glycation modifications depicting surface exposed lysine residues (A) glycated BSA with aminoguanidine hydrochloride (B) glycated BSA with eugenol methyl ether

(A)

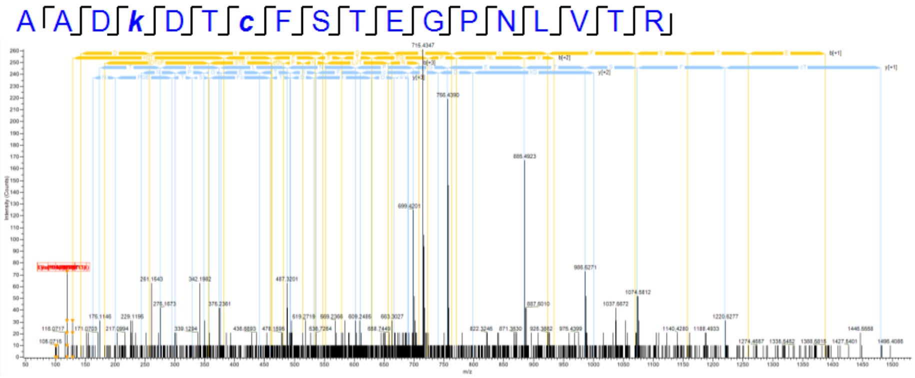

AAD**K**DT**C**FSTEGPNLVTR Amadori K(4), Carbamidomethyl C(7)

(B)

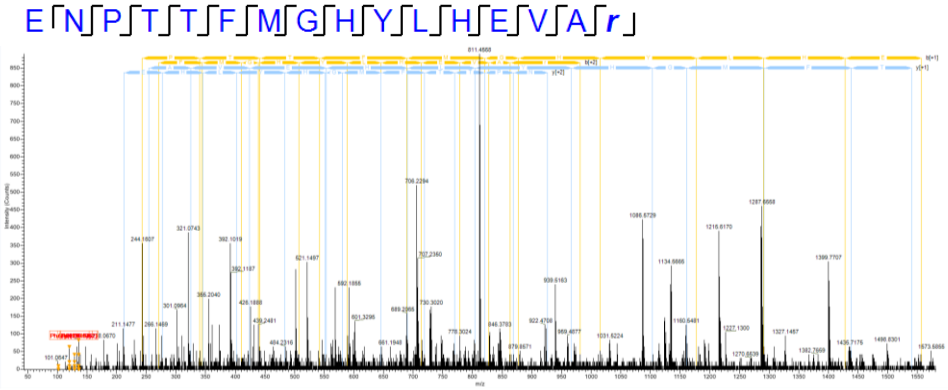

ENPTTFMGHYLHEVA**R** Amadori R(16)

(C)

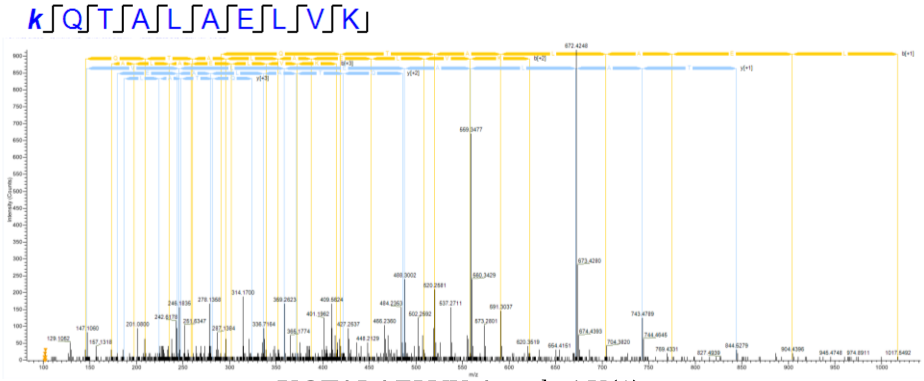

KQTALAELVK Amadori K(1)

(D)

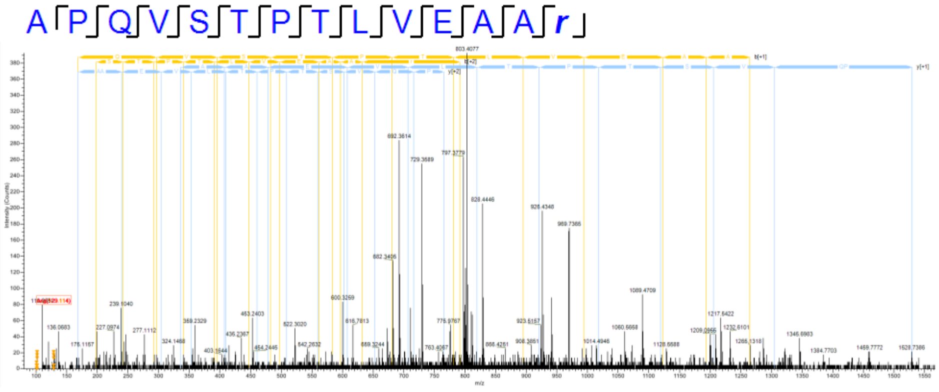

APQVSTPTLVEAA**R** Amadori R (14)

Supplementary figure S2: MS/MS spectra annotation of AGE modified peptides of MSA at (A) K588 (B) R168 (C) K549 (D) R452; K, Lysine; R, Arginine.

(A)

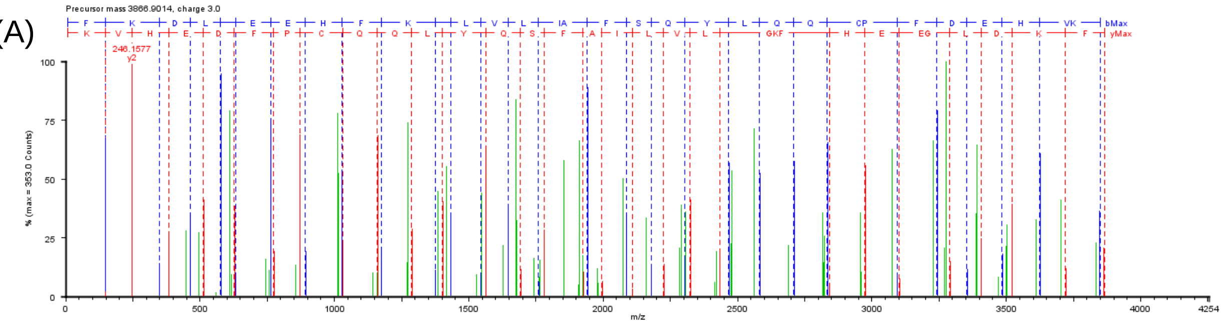

(R)FKDLGEEHFKGLVLIAFSQYLQQCPFDEHVK(L) CEL (2), CEL (10), Carbamidomethyl C (24)

(B)

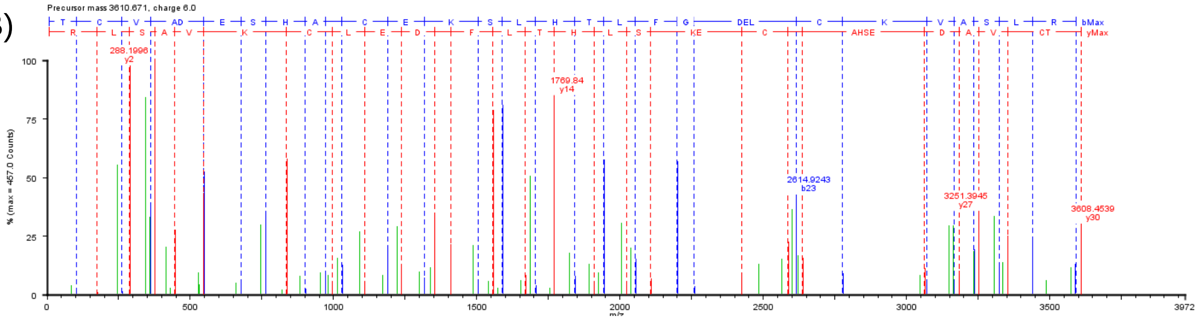

(K)TCVADESHAGCEKSLHTLFGDELCKVASLR(E) Carbamidomethyl C (2), Carbamidomethyl C (11), CML (13), Carbamidomethyl C (24), AMADORI (25)

(C)

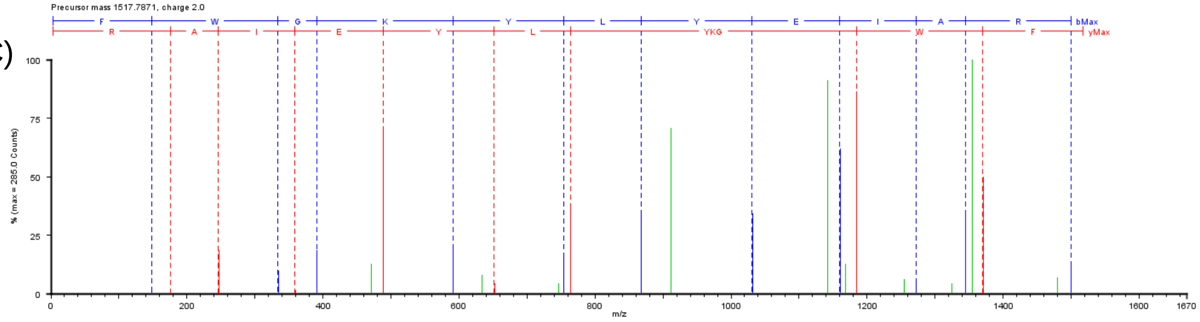

(K)FWGKYLYEIAR(R) CEL (4)

(D)

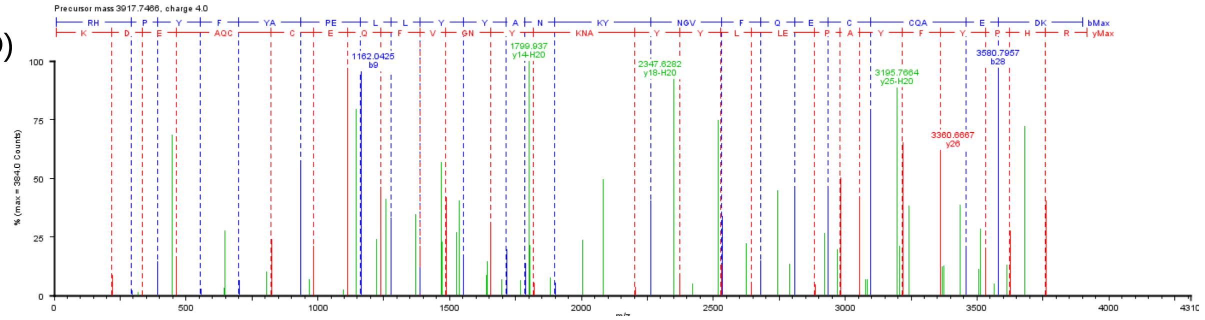

(R)RHPYFYAPELLYYANKYNGVFQECCQAEDK(G) CEL (16), Carbamidomethyl C (24), Carbamidomethyl C (25), CEL (30)

(E)

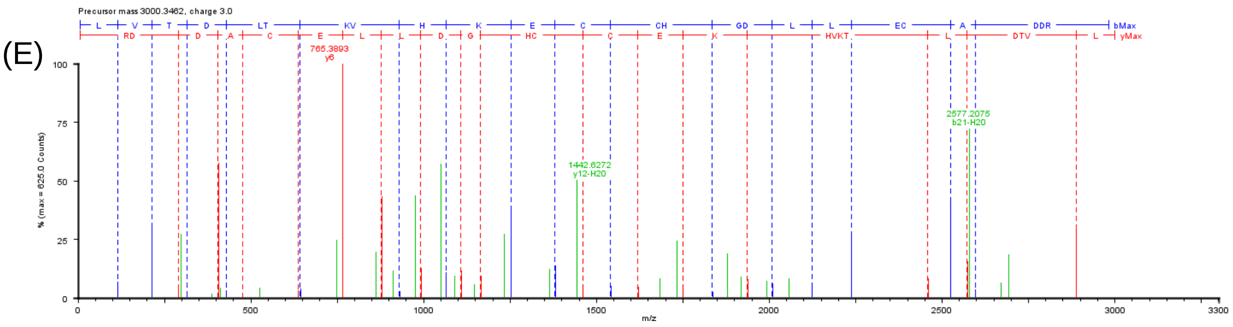

(K)LVTDLTKVHKECCHGDLLECADDR(A) CML (7), CML (10), Carbamidomethyl C (12), Carbamidomethyl C (13), Carbamidomethyl C (20)

(F)

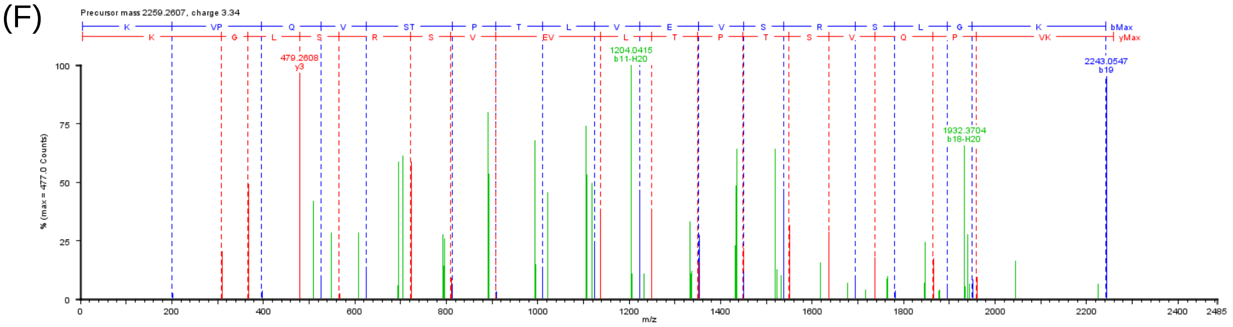

(R)KVPQVSTPTLVEVSRSLGK(V) CEL (1), AMADORI (19)

(G)

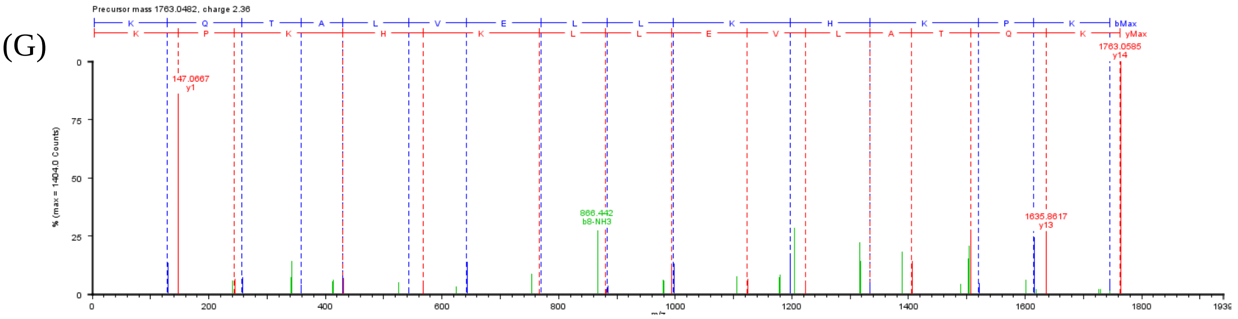

(K)KQTALVELLKHKPK(A) CEL (10), CML (12)

Supplementary Figure S3: MS/MS spectra annotation of AGE modified peptides of BSA at (A) K36 (B) K88 (C) K160 (D) K184 (E) K263 (F) K438 (G) K548

Supplementary Table S1: GC-MS-based chemical profiling of leaf tissue of *Ocimum kilimandscharicum, Ocimum tenuiflorum* and *Ocimum gratissimum*

Compound name	*O. kilimandscharicum*	*O. tenuiflorum*	*O. gratissimum*
Monoterpenes			
Pinene	0.92 ± 0.2	ND	ND
β- Ocimene	ND	ND	4.94 ± 0.3
Borneal	0.74 ± 0.003	ND	ND
Borneol	ND	1.2 ± 0.06	ND
Camphene	3.59 ± 0.01	ND	ND
Camphor	**47.33 ± 0.3**	ND	ND
Eucalyptol	19.85 ± 0.16	ND	ND
Limonene	4.97 ± 0.01	ND	ND
Myrtenol	0.99 ± 0.15	ND	ND
Terpineal	0.28 ± 0.002	ND	ND
Terpineol	0.25 ± 0.03	ND	ND
Terpinolene	0.41 ± 0.01	ND	ND
Thujanol	2.78 ± 0.01	ND	0.5
Cis-thujene	ND	ND	0.24
Sesquiterpenes			
α- Caryophyllene	0.45 ± 0.02	ND	ND
α- Copaene	0.56 ± 0.01	3.33 ± 0.12	1.54
α- Humulene	ND	0.88 ± 0.04	ND
β- Bourbonene	ND	1 ± 0.05	0.39
β- Caryophyllene	3.68 ± 0.02	14.5 ± 0.09	2.87 ± 0.03
β- Cubebene	0.39 ± 0.004	2.33 ± 0.01	0.66 ± 0.05
β- Elemene	0.25 ± 0.002	0.89 ± 0.06	0.36 ± 0.05
δ- Cadinene	0.18 ± 0.01	3.11 ± 0.15	0.36 ± 0.001
Elemol	ND	1.72 ± 0.08	ND
Farnesene	0.69 ± 0.23	ND	ND
Germacrene D	5.19 ± 0.05	5.83 ± 0.35	9.52 ± 0.15
Germacrene-D-al	0.09 ± 0.01	ND	0.21
Murrolene	ND	ND	0.10 ± 0.01
Others			
Eugenol	ND	ND	**78.25 ± 0.4**
Eugenol Methyl Ether	ND	**60.41 ± 0.75**	ND
Dodecane	0.19 ± 0.002	0.37± 0.02	ND
Dodecene	0.18 ± 0.002	0.43 ± 0.01	ND
Heptene	0.33 ± 0.02	ND	ND
Octane derivative	0.10 ± 0.03	0.46 ± 0.12	ND
Tetradecane	ND	0.23 ± 0.01	ND

^ND (not detected)

Suplementary Table S2: NMR spectroscopic data of purified compounds

**Compound (Mol. Wt.)**	**Purity (%)**	**^{1}H NMR**	**^{13}C NMR**
Eugenol<br><br>(164 g/mol)	99.9	(200 MHz, $CDCl_3$); δ(ppm): 3.30–3.33(d, 2H, H-7), 3.87 (s, 3H, H-10), 5.03 (br s, 1H, H-9), 5.08 -5.12 (m, 1H, H-9), 5.5 (br s, 1H, 1-OH), 5.85-6.05 (m, 1H, H-8), 6.69 (m, 2H, H-3, 5), 6.83-6.87 (m, 1H, H-6).	(50 MHz, $CDCl_3$); δ(ppm): 39.86 (C-7), 52.82 (C-10), 111.05 (C-3), 114.20 (C-6), 115.49 (C-9), 121.14 (C-5), 131.89 (C-4), 137.79 (C-8), 143.86 (C-1), 146.39 (C-2)
Eugenol Methyl Ether<br><br>(178 g/mol)	98.2	(200 MHz, $CDCl_3$); δ(ppm): 3.85 (s, 3H, H-10), 3.86 (s, 3H, H-11), 3.31-3.34 (d, 2H, H-7), 5.03 (br s, 1H, H-9), 5.08 (m, 1H, H-9), 5.85-6.05 (m, 1H, H-8), 6.70 (br s, 1H, H-3), 6.73-6.74 (m, 1H, H-5), 6.78-6.82 (m, 1H, H-6).	(50 MHz, $CDCl_3$); δ(ppm): 39.77 (C-7), 55.75 (C-10), 55.89 (C-11), 111.18 (C-3), 111.79 (C-6), 115.57 (C-9), 120.35 (C-5), 132.59 (C-4), 137.66 (C-8), 147.32 (C-1), 148.84 (C-2).
Camphor<br><br>(152 g/mol)	98	(200 MHz, $CDCl_3$); δ(ppm): 2.29-2.42 (m, 1H, H-3), 0.84 (s, 3H, H-8), 0.91 (s, 3H, H-9), 0.96 (s, 3H, H-10), 1.25-1.47 (m, 2H, H-4), 1.65-1.80 (m, 2H, H-5), 1.89-2.09 (m, 2H, H-2).	(50 MHz, $CDCl_3$); δ(ppm): 9.22 (C-10), 19.12 (C-8), 19.76 (C-9), 27.02 (C-4), 29.89 (C-5), 43.02 (C-3), 43.28 (C-2), 46.77 (C-7), 57.69 (C-6), 200.98 (C-1).

Supplementary Table S3: Average precursor ion intensity and other information of AGE modified and corresponding unmodified peptides in *in vivo* plasma samples.

Sl	Mod Site	Peptide Start-end	Peptide sequence	Peptide m/z Da	Peptide MH+ Da	PCS	Avg. XCorr	STZ-control (APII)	Vehicle control (APII)	Eug (APII)
1	588	585-602	AAD**K***DT**C***FSTEGPNLVTR	715.3	2143.97	+3	4.44	2.29e4	1.60e4	2.24e4
2	UM	585-602	AADKDTCFSTEGPNLVTR	661.3	1981.92	+3	6.39	2.44e4	5.81e5	4.41e5
3	168	153-168	ENPTTFMGHYLHEVA**R***	688.6	2063.95	+3	2.10	2.06e4	2.20e4	7.88e3
4	UM	153-168	ENPTTFMGHYLHEVAR	634.6	1901.90	+3	5.16	2.16e4	3.10e5	9.20e4
5	549	549-558	**K***QTALAELVK	421.5	1262.71	+3	3.71	1.20e5	8.41e4	5.78e4
6	UM	549-558	KQTALAELVK	367.5	1100.66	+3	4.72	3.51e5	7.05e5	7.78e5
7	452	439-452	APQVSTPTLVEAA**R***	801.4	1601.83	+2	1.03	1.49e4	8.56e4	8.92e4
8	UM	439-452	APQVSTPTLVEAAR	720.4	1439.79	+2	4.46	1.5e4	9.64e4	1.50e5

^ UM, Unmodified; * Indicates modified amino acid side chain; PCS, Peptide Charge State; APII, Average Precursor Ion Intensity.

Supplementary Table S4: Extent of AGE modification on peptides *in vitro*. Values in the table represent the average cumulative intensity ratio (CIR) of AGE modified peptides to their unmodified form

Sample/Site	K36	K88	K160	K184	K263	K438	K490	K548
BSA	0 (± 0.0)	0 (± 0.0)	0 (± 0.0)	0 (± 0.0)	0 (± 0.0)	0 (± 0.0)	0 (± 0.0)	0 (± 0.0)
glycated BSA	11.91 (± 02.56)	14.52 (±02.32)	47.09 (±10.78)	16.36 (±04.40)	12.70 (±01.61)	184.28 (± 46.10)	09.44 (±02.47)	199.82 (±47.55)
glycated BSA+AMG	04.91 (± 00.83)	06.93 (±00.52)	16.46 (±03.63)	06.07 (±01.08)	05.46 (±00.56)	68.79 (± 14.04)	03.47 (±00.43)	30.61 (±03.44)
glycated BSA+EUG	02.03 (± 00.11)	03.43 (±00.69)	05.90 (±00.96)	04.08 (±00.23)	06.19 (±01.32)	24.66 (± 06.62)	05.47 (±01.08)	36.52 (±03.20)

^ Bovine Serum Albumin, BSA; Aminoguanidine hydrochloride, AMG; Eugenol, EUG; lysine, K; Arginine, R.
